# *LncRNA29RIK* in macrophages promotes *LPS-*mediated sensitivity to obesity

**DOI:** 10.3389/fimmu.2025.1574507

**Published:** 2025-04-28

**Authors:** Rong Wang, Yunhuan Gao, Ya Wang, Yuan Zhang, Rongcun Yang

**Affiliations:** ^1^ Department of Immunology, Nankai University School of Medicine, Nankai University, Tianjin, China; ^2^ Translational Medicine Institute, Affiliated Tianjin Union Medical Center of Nankai University, Tianjin, China; ^3^ State Key Laboratory of Medicinal Chemical Biology, Nankai University, Tianjin, China

**Keywords:** obesity, macrophages, *lncRNARIK*, caspase 4, pyroptosis

## Abstract

Lipopolysaccharide (LPS, endotoxin) -mediated signaling of caspase-4 (human) and -11 (rodent) can induce the maturation of inflammatory cytokine IL-1β and cell pyroptosis, which is associated with the pathophysiology of many diseases such as obesity. However, the process by which LPS induces inflammation through caspase 4/11 is not fully understood. We found here that *lncRNA29RIK* plays a key role in LPS-mediated maturation of inflammatory cytokine IL-1β and pyroptosis of macrophages. Mechanistic ally, the binding of caspase 4/11 to LPS requires *lncRNARIK* to cause activation of the caspase 4/11 complex, which ultimately caused inflammation to promote sensitivity to high fat diet (HFD) -mediated obesity. Notably, *lncRNA29RIK* expression can be up-regulated by LPS. This lncRNA29 is highly conserved between humans and mice. Taken together, these results suggest that *lncRNA29RIK* determines the occurrence and progression of LPS-related diseases such as obesity.

## 1 Introduction

Low-grade inflammation is a frequent feature of metabolic diseases such as obesity (1). LPS has been identified as a key contributing factor in the initiation and progression of the inflammation (2). It can activate macrophages in tissues such as adipose tissues to induce inflammation (3). Macrophages, as principal phagocytic components of the immune system, are among the primary regulators of innate immunity responsible for a broad range of inflammatory processes (4).

10LPS-mediated human caspase-4 and mouse caspase-11 (caspase-4/11) signaling can cause maturation of inflammatory cytokine IL-1β and cytopyroptosis of macrophages. The pyroptosis is executed by the pore-forming protein gasdermin-D (GSDMD), which is activated by cleavage mediated by caspase-4/11. The active caspase-4/11 can cleave GSDMD, which forms GSDMD pore (pyroptosis) to cause NLRP3 activation (5). Activation of NLRP3 inflammasomes elicits caspase-1 cleavage, which can induce interleukin-1β (IL-1β) production (6). Notably, caspase-4/11 activation in macrophages with LPS or Gram-negative bacteria requires the expression of interferon (IFN)-inducible guanosine triphosphate (GTP)ases, such as guanylate-binding proteins (GBPs) and/or immunity-related GTPases (IRGs) (7–10). A high molecular weight complex formed by GBP and LPS could promote the recruitment of caspase-4/11 and subsequently transfer LPS onto caspase-4/11 to trigger its activation (11). The macrophages lacking GBPs show impaired caspase-4/11 activation and attenuated pyroptosis (7). However, it is not clear how this process, GBP and LPS mediated the recruitment of caspase-4/11, occurs.

Non-coding RNAs (ncRNAs) are a class of RNA transcripts lacking the ability to encode peptides or proteins. These lncRNAs are now recognized as playing crucial roles in numerous cellular processes, including the cell cycle, differentiation, and metabolism, and in disease. In the cytoplasm, lncRNAs can function to mediate signal transduction pathways, translational programs, and posttranscriptional control of gene expression. For example, a recent study revealed that lncRNAs serve as scaffolds in the cytoplasm to nucleate complex networks of proteins functioning in regulating signaling transduction programs, such as the Toll-like receptor/TIR-domain containing adapter-inducing IFN-β (TLR-TRIF) immune pathway (12). Based on the ubiquity of protein–RNA interactions, many studies have emphasized how their perturbations are related to pathology, including autoimmune diseases, neurological diseases, and cancer (13). We here found that that LPS-mediated LncRNA *lncRNA29RIK* in macrophages can promote the oligomerization of the LPS/caspase-4/11 complexes upon exposure to LPS/dotap, which can cause tissue inflammation and promote sensitivity to obesity through the LPS-mediated release of mature (m) IL-1β and pyroptosis of macrophages.

## 2 Materials and methods

Reagents and oligoes used in this study are listed in **Supplementary Table S1**.

### 2.1 Mice and cell lines

*LncRNA29Rik*-deficient mice on a C57BL/6J background were generated by the Model Animal Research Center of Nanjing University (Nanjing, Jiangsu, China) using CRISPR-Cas9 system as previously reported by us (14, 15). Caspase1/11 knockout (KO) mice were from Prof. Shao, National Institute of Biological Sciences, Beijing. All mice were maintained under specific pathogen-free (SPF) conditions in the Animal Center of Nankai University. All animal experiments were approved (Ethic approval no: NK-202019) and carried out in accordance with Nankai University Guide for the Care and Use of Laboratory Animals. Human embryonic kidney cell line HEK 293T cells were obtained from the American Type Culture Collection.

### 2.2 Mouse models

For high-fat diet (HFD) model, 6-8-week-old male mice and their control littermates were fed using HFD (D12492; protein, 26.2%; carbohydrate, 26.3%; and fat, 34.9%) and control diets [D12450B (60% of calories may be derived from fat)], which was from Research Diets, Inc. (NJ, USA).

For *Salmonella typhimurium* (ATCC14028) infection, *Salmonella* infection model was performed according to the previous method (16). Briefly, mice were withdrawn from water and food for 4 h before oral gavage treatment. Then, mice were treated with 7.5 mg of streptomycin. At 20 h after streptomycin treatment, mice were withdrawn from water and food again and then infected with *S. typhimurium* (200 cfu). Mice were weighed every other day for the determination of percent weight change. This was calculated as: % weight change = (weight at day X − day 0/weight at day 0) × 100.

For toxic experiment, mice were intraperitoneally injected with 54 mg/kg LPS (O111:B4, Sigma), survival (time to moribund) were detected, and then, serum concentration of IL-1β were detected.

### 2.3 Preparation of macrophages

For macrophages from peritoneal cavity of mice, macrophages were generated in the peritoneal cavity of mice by intraperitoneally injecting with 4 mL of 3% thioglycollate medium. After 4 days, 5 mL of cold phosphate-buffered saline (PBS) containing 3% FBS was injected into the peritoneal cavity. Following this injection, a gentle massage was performed, and peritoneal fluid was subsequently isolated. Next, cells derived from the peritoneal washing fluid were seeded at 2 × 10^6^ in RPMI containing 10% FBS. Non-adherent cells were removed 4 h after seeding by extensive washing with medium.

For human monocyte-derived macrophages (HMDM), primary human peripheral blood mononuclear cells (PBMCs) were isolated by Ficoll–Hypaque density gradient centrifugation. CD14^+^ magnetic isolation kit was used to isolate monocytes/macrophages following the manufacturer’s instructions. Monocytes/macrophages were cultured in DMEM with 10% FBS, 50 ng/mL human M-CSF, and 1% penicillin/streptomycin for 4 days and then used for experiments.

For THP1-derived macrophages (THP), THP-1 cells were treated 24 h with 100 ng/mL PMA. For mouse bone-marrow-derived macrophages (BMDMs), BMDMs were obtained from the bone marrow of the tibia and femur and cultured in DMEM with 10% FBS, 20 ng/ml mouse M-CSF, and 1% penicillin/streptomycin for 6 days and then used for experiments.

### 2.4 Macrophage stimulation

For macrophages stimulation, macrophages were primed by priming with 2 μg/mL LPS for 4 h followed by treatment with 5 µM nigericin and 2 μg/mL LPS using Dotap transfection reagent for 30 min. THP-1 cells were treated 24 h with 100 ng/mL PMA before overnight stimulation. Then, supernatants were analyzed for IL-1β by ELISA and LDH by LDH detecting kit.

### 2.5 Metabolism experiments

For glucose and insulin tolerance, baseline blood glucose levels were measured using a Nova Max Plus GlucoseMeter after 5 h of fasting. Mice were then injected intraperitoneally with 2 g glucose per kg body weight in sterile PBS or with 0.5 U insulin per kg body weight, and blood glucose levels were measured at different times after injection.

### 2.6 Transfection of microRNA, preparation of plasmids, and construction and transduction of shRNA or *lncRNA29RIK* lentiviruses

For microRNA transfection, peritoneal macrophages were transfected with microRNAs using HiPerFect transfection reagent according to the manufacturer’s instructions. For preparation of plasmids, the sequences or fragments of mouse caspase-11, human caspase-4, and mouse/human *lncRNA29RIK* were amplified using PCR methods. The PCR products were cloned into the pcDNA™3.1/V5-His TOPO^®^ TA plasmid (Invitrogen). After sequencing, plasmid constructions were used to transfect HEK 293T cells. For lentivirus construction and transduction, a short hairpin RNA (shRNA) target sequence was chosen by BLOCK-iT™ RNAi Designer (Invitrogen). The constructs were made using pGreenPuro™ cloning and expression lentivector kit (System Biosciences Inc.) according to the manual. The negative control (NC) is luciferase control RNA from the kit. For packaging lentivirus particles, lentivector together with pMD2.G and psPAX2 packaging plasmids were co-transfected into 293T cells. Peritoneal macrophages were infected with the lentiviral supernatants in the presence of 8 μg/ml polybrene (Millipore) by centrifugation and then cultured with complete medium for 24 h.

### 2.7 RNA extraction and qRT-PCR

RNA extraction and qRT-PCR were analyzed according to our previously reported methods (15, 17). The fold changes were calculated using the ΔΔCt method according to the manufacturer’s instructions (Applied Biosystems). All the reactions were run in triplicate.

### 2.8 Cytosolic and nuclear fractionation

Indicated cells were incubated with hypotonic buffer (25 mM Tris–HCl, pH 7.4, 1 mM MgCl_2_, 5 mM KCl) on ice for 5 min. An equal volume of hypotonic buffer containing 1% NP-40 was then added, and each sample was left on ice for another 5 min. After centrifugation at 5,000 × g for 5 min, the supernatant was collected as the cytosolic fraction. The pellets were resuspended in nucleus resuspension buffer (20 mM HEPES, pH 7.9, 400 mM NaCl, 1 mM EDTA, 1 mM EGTA, 1 mM DTT, and 1 mM PMSF) and incubated at 4°C for 30 min. Nuclear fraction was collected after removing insoluble membrane debris by centrifugation at 12,000*g* for 10 min.

### 2.9 H&E staining

For hematoxylin/eosin (H&E) staining, previously reported methods were used in this experiment (15, 17, 18). Briefly, lung tissues were fixed in 4% (w/v) paraformaldehyde-buffered saline and embedded in paraffin; 5-µm colon sections were cut and stained with H&E.

### 2.10 Cell isolation and flow cytometry

Previous reported protocol was used in cell isolation and flow cytometry (19). Briefly, for the staining of lamina propria (LP) lymphocytes, the gut was isolated and cleaned by shaking in ice-cold PBS four times before the tissue was cut into 1-cm pieces. The epithelial cells were removed by incubating the tissue in HBSS with 2 mM EDTA for 30 min at 37°C with shaking. LP cells were isolated by incubating the tissues in digestion buffer [DMEM, 5% fetal bovine serum, 1 mg/mL collagenase IV (Sigma-Aldrich) and DNase I (Sigma-Aldrich, Tianjin, P. R. China)] for 40 min. The digested tissues were then filtered through a 40-mm filter. Cells were resuspended in 10 ml of the 40% fraction of a 40:80 Percoll gradient and overlaid on 5 mL of the 80% fraction in a 15-mL Falcon tube. Percoll gradient separation was performed by centrifugation for 20 min at 1,800 rpm at room temperature. LP cells were collected at the interphase of the Percoll gradient, washed and resuspended in medium, and then stained and analyzed by flow cytometry. Dead cells were eliminated through 7-AAD staining.

For the staining of immune cells in adipose tissues, adipose tissues first were cut into smaller pieces and then digested in digestion buffer (20 μL/mL collagenase I for 35 min. The digested tissues were then filtered through a 40-mm filter.

### 2.11 Statistical analyses

Two-sided Student’s *t*-test and one-way ANOVA Bonferroni’s multiple comparison test were used to determine significance. The statistical significance of the survival curves was estimated using Kaplan and Meier method, and the curves were compared using the generalized Wilcoxon’s test. These were performed by GraphPad Prism 5 software (GraphPad Software). A 95% confidence interval was considered significant and was defined as *p* < 0.05.

### 2.12 Others

For Western blot and immunoprecipitation, previously reported methods were used in this study (15, 17, 18). RNA immunoprecipitation was performed according to previously reported protocol (15, 17). For immunostaining and RNA-FISH, previously reported protocols were used (15, 17, 18). For biotin-labeled LPS pull-down analyses, the biotin-labeled LPS and streptavidin magnetic beads were added into the cell lyses and incubated for 4 h. After centrifuge, the precipitations were analyzed using immunoblotting. For gene binding motif analyses, sequence logo of gene binding motif was obtained used the MEME software (https://meme-suite.org/meme/) (lower). NCBI database was used to perform sequence comparison to find out homologous sequences.

## 3 Results

### 3.1 LPS promotes the expression of *lncRNA29RIK*

**Figure 1:**
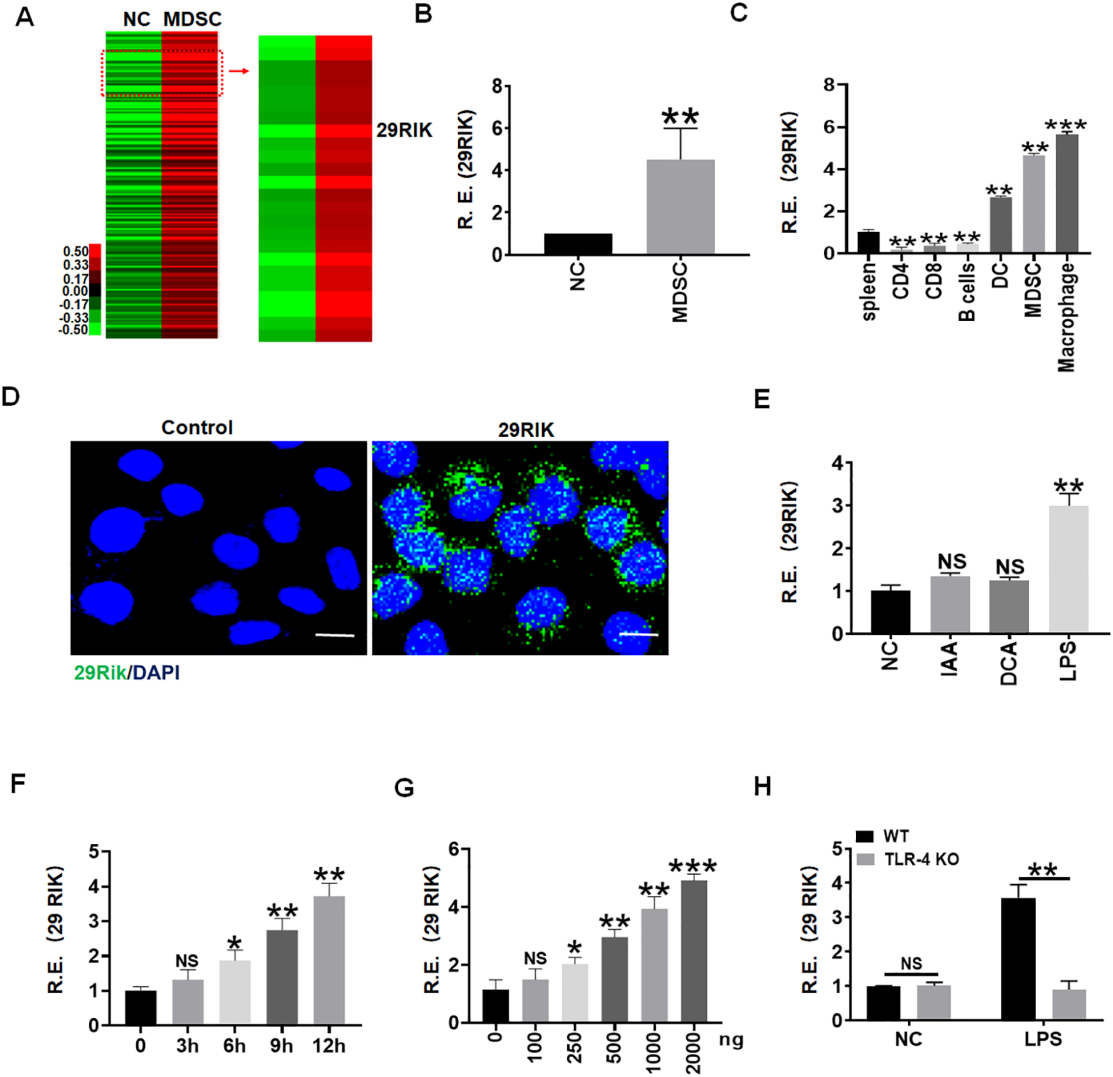
LPS promotes expression of Lnc29rik (29RIK) in macrophages. **(A)** LncRNA microarray of myeloid-derived suppressor cells (MDSCs). MDSCs were generated, and lncRNA expression was then evaluated using an lncRNA expression microarray. NC, bone marrow cells. **(B)** QRT-PCR of *lncRNA29RIK* in MDSCs. NC, bone marrow cells. R. E, relative expression. **(C)** qRT-PCR of *lncRNA29RIK* in the spleen, and CD4, CD8, B cell, dendritic cells (DC), MDSC, and macrophages sorted from the spleen by flow cytometry. R. E, relative expression. **(D)** Fluorescence *in situ* hybridization of *lncRNA29RIK* in mouse macrophage upon exposure to LPS (1 μg/mL) for 24 h Nuclei were stained with DAPI (blue); green, *lncRNA29RIK*. Scale bar, 2.5 μM. **(E)** qRT-PCR of *lncRNA29RIK* in the macrophages after exposure to IAA, DCA, or LPS for 24 h **(F)** qRT-PCR of *lncRNA29RIK* in the macrophages at the different times after exposure to LPS (1 μg/mL). R. E, relative expression. **(G)** qRT-PCR of *lncRNA29RIK* in the macrophages after exposure to different concentrations of LPS. R. E, relative expression. **(H)** QRT-PCR of *lncRNA29RIK* in the TLR4 KO macrophages. Ctr., bone marrow cells. R. E, relative expression. One-way ANOVA Bonferroni’s multiple comparison test used in parts **C** and **E-G** and Student’s *t*-test in parts **B** and **H**; *p < 0.05, **p < 0.01, ***p < 0.001; NS, not significant.

While we investigated gene expression in the myeloid derived cells (MDSCs) generated by co-culturing bone marrow cells with GM-CSF and IL-6 for 4 days, we found that lncRNA *lncRNA29RIK* could not only express in mouse MDSCs but also in macrophages (**Figures 1A-D**). However, there were only low levels of expression in other immune cells such as CD4, CD8, and B cells (**Figure 1C**). Since gut microbiota plays a critical role in the differentiation and function of immune cells through genetic and epigenetic modification (20, 21), we explored the gut-microbiota-derived component(s) to regulate the expression of epigenetic modification factor *lncRNA29rik* in the macrophages. We screened the effects of gut microbiota components and metabolites such as short-chain fat acids (SCFAs) and bile-acid- and tryptophan-derived metabolites on the macrophages, which were generated in the peritoneal cavity of mice by intraperitoneal injection of thioglycolate medium. Interestingly, *lncRNA29rik* expression could be regulated by Gram-negative bacterium LPS but not by other gut-microbiota-derived metabolites such as indole acetic acid (IAA) and deoxycholic acid (DCA) (**Figure 1E**). Increased expression of *lncRNA29rik* were dose and time dependent in the macrophages after exposure to LPS (**Figures 1F,G**). Previous studies showed that LPS-mediated activation of macrophages was dependent on TLR4 (22). Indeed, LPS could induce the expression of *lncRNA29RIK* in the wild-type (WT) but not in TLR4 knockout (KO) macrophages. *LncRNA29rik* or Kantr in mice and KANTR in humans were located on chromosome X, as shown in **Supplementary Figure S1**. After sequence similarity analysis, it was found that this lncRNA had 78% homology between mice and humans (**Supplementary Figure S1**).

### 3.2 *LncRNA29RIK* promotes the production of mature IL-1β and pyroptosis in macrophages

**Figure 2:**
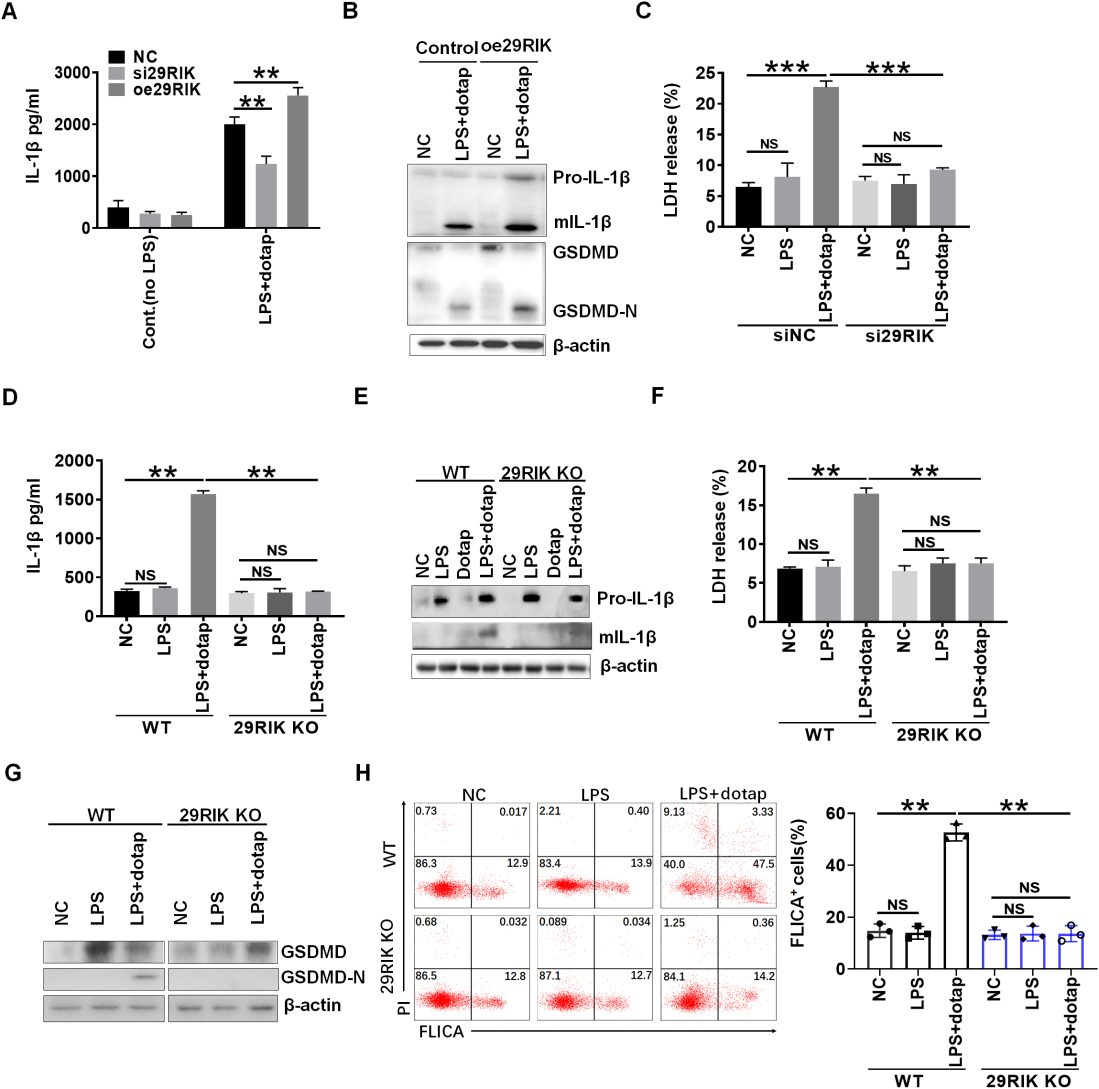
*LncRNA29RIK* (29RIK) affects mature IL-1β and pyroptosis in the macrophages. **(A)** ELISA of IL-1β in the supernatants of *lncRNA29RIK siRNA* (si29RIK) or exogenous *lncRNA29RIK* (oe29RIK) transfected macrophages after exposure to LPS/dotap. NC, control. **(B)** Immunoblotting of pro-IL-1β, mature (m) IL-1β, GSDMD, and cleaved-GSDMD (GSDMD-N) in exogenous *lncRNA29RIK* (oe29Rik) transfected macrophages after exposure to LPS/dotap. NC, no stimulator. **(C)** Analyses of LDH in the supernatants of *lncRNA29RIK* siRNA (si29RIK) transfected macrophages after exposure to LPS/dotap. siNC, siRNA control; NC, no stimulator. **(D)** ELISA of IL-1β in the supernatants of *lncRNA29RIK* KO (29RIK KO) macrophages after exposure to LPS/dotap. NC, control. **(E)** Immunoblotting of pro-IL-1β and mIL-1β in *lncRNA29RIK* KO macrophages after exposure to LPS/dotap. NC, control. **(F)** Analyses of LDH in the supernatants of *lncRNA29RIK* KO macrophages after exposure to LPS/dotap. NC, control. **(G)** Immunoblotting of GSDMD and cleaved-GSDMD (GSDMD-N) in *lncRNA29RIK* KO macrophages after exposure to LPS/dotap.NC, control. **(H)** flow cytometry of *lncRNA29RIK* KO macrophages after FLICA staining on exposure to LPS/dotap; NC, control. Student’s *t*-test; *p < 0.05, **p < 0.01, ***p < 0.001; NS, not significant.

We next analyzed the function of mouse *lncRNA29RIK* (*mlncRNA29RIK*) in macrophages. Silencing *mlncRNA29RIK* could significantly decrease production of mature IL-1β (mIL-1β), whereas increased mIL-1β could be observed in *mlncRNA29RIK* overexpressing macrophages after exposing caspase-4/11 ligand, LPS with Dotap (LPS/dotap) (23) (**Figures 2A, B**). The production of mIL-1β often companies with pyroptosis (24, 25), which is a lytic cell death induced by pathogen infection or endogenous challenge (26). Indeed, caspase-4/11 ligand LPS/dotap also induced more lactate dehydrogenase (LDH) release in the monocytes/macrophages but not in *mlncRNA29RIK* silencing cells after exposure to LPS/dotap (**Figure 2C**), suggesting that the pyroptosis of monocytes/macrophages was dependent on *mlncRNA29RIK*. Indeed, there were more macrophage pyroptosis in *mlncRNA29RIK* overexpressing macrophages after exposure to LPS/dotap, whereas there were less macrophage pyroptosis in *lncRNA29RIK* silencing cells as compared to their controls (**Supplementary Figure S2**).

To further determine the function of *mlncRNA29RIK*, we generated *mlncRNA29RIK* knockout (KO) mice. The *mlncRNA29RIK* KO macrophages were generated in the peritoneal cavity of mice by intraperitoneal injection with thioglycolate medium. There was no difference in the production of mIL-1β and pyroptosis in *mlncRNA29RIK* KO macrophages with and without exposing to caspase-11 ligands LPS/Dotap, whereas markedly increased production of mIL-1β and pyroptosis could be detected in the macrophages from WT mice (**Figures 2D–G**), indicating that *mlncRNA29RIK* plays a critical role in production of mIL-1β and pyroptosis of monocytes/macrophages. *MlncRNA29RIK-*mediated pyroptosis of monocytes/macrophages were further confirmed by flow cytometry through FLICA staining (**Figure 2H**). Notably, *mlncRNA29RIK* KO did not significantly change the expression of cytokines and co-stimulation and also differentiation of myeloid derived cells (**Supplementary Figure S3**). Taken together, these results indicate that *mlncRNA29RIK* play a role in LPS-mediated production of mIL-1β and macrophage pyroptosis.

### 3.3 *LncRNA29RIK* KO reduces sensitivity of mice to high-fat </b><b>diet-mediated obesity

**Figure 3:**
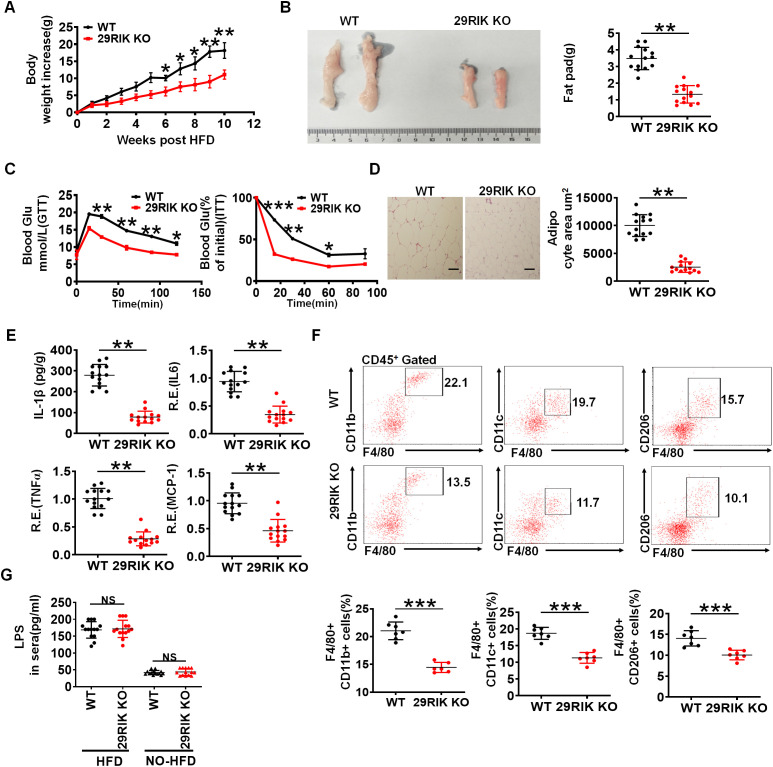
*LncRNA29RIK* (29RIK) KO mice are resistance to HFD-mediated obesity. **(A)** Body weight increases in male WT and *lncRNA29RIK* KO mice fed high-fat diet (HFD) (n=14). These mice are no differences at baseline before feeding HFD. **(B)** Fat-pad in male WT and *lncRNA29RIK* KO mice fed HFD for 3 months. **(C)** Glucose tolerance and insulin sensitivity of WT and *lncRNA29RIK* KO mice fed HFD for 3 months (n=6). **(D)** H/E staining of adipose tissues of WT and *lncRNA29RIK* KO mice fed HFD. One representative. **(E)** ELISA of mIL-1β and qRT-PCR of TNFα, IL-6 and MCP-1 in the adipose tissues of WT and *lncRNA29RIK* KO mice fed HFD (n=14). **(F)** Flow cytometry of F4/80 CD11b. F4/80 CD11C and F4/80 CD206 in adipose tissues of WT and *lncRNA29RIK* KO mice fed HFD. **(G)** Concentration of LPS in the sera of WT and *lncRNA29RIK* KO mice fed HFD (n=14). Analysis of variance test in parts **(A, C)**; Student’s *t*-test in other panels, mean ± SD. R. E, relative expression. *p < 0.05, **p < 0.01, and ***p < 0.001. Data are a representative of three independent experiments.

Obesity is strictly a relationship with LPS-mediated inflammatory macrophages in adipose tissues (27, 28). The high-fat diet (HFD) can induce the disruption of the intestinal barrier, which cause the release of bacterial metabolites and endotoxins, such as LPS into the circulation (27). Since LPS-mediated *lncRNA29RIK* could promote the production of IL-1β in the macrophages, we next employed HFD-mediated obesity model to detect the effects of *lncRNA29RIK* KO on the obesity. While mice were fed with HFD, *lncRNA29RIK* KO mice showed marked resistance to HFD-induced obesity, including less body weight and weight of fat-pad tissues, increased sensitivity to insulin, and tolerance to glucose (**Figures 3A–C**). Histochemical staining showed less adipose cells in *lncRNA29RIK* KO mice than WT mice (**Figure 3D**). mIL-1β was also much lower in the adipose tissues of *lncRNA29RIK* KO mice than control WT mice (**Figure 3E**). Inflammatory cytokines TNFα, IL-6, and MCP-1 were also markedly decreased in in the adipose tissues of *lncRNA29RIK* KO mice (**Figure 3E**). There were also less inflammatory macrophages (F4/80/CD11b, F4/80/CD11c and F4/80/TNFα cells) in the adipose tissues of *lncRNA29RIK* KO mice (**Figure 3F**). Consistent with other reports, there were also higher levels of LPS in the sera of mice fed HFD as compared to control mice fed normal diet (**Figure 3G**). Taken together, *lncRNA29RIK* KO reduces sensitivity of mice to HFD-mediated obesity.

### 3.4 *LncRNA29RIK* promotes binding of caspase-11 with LPS

**Figure 4:**
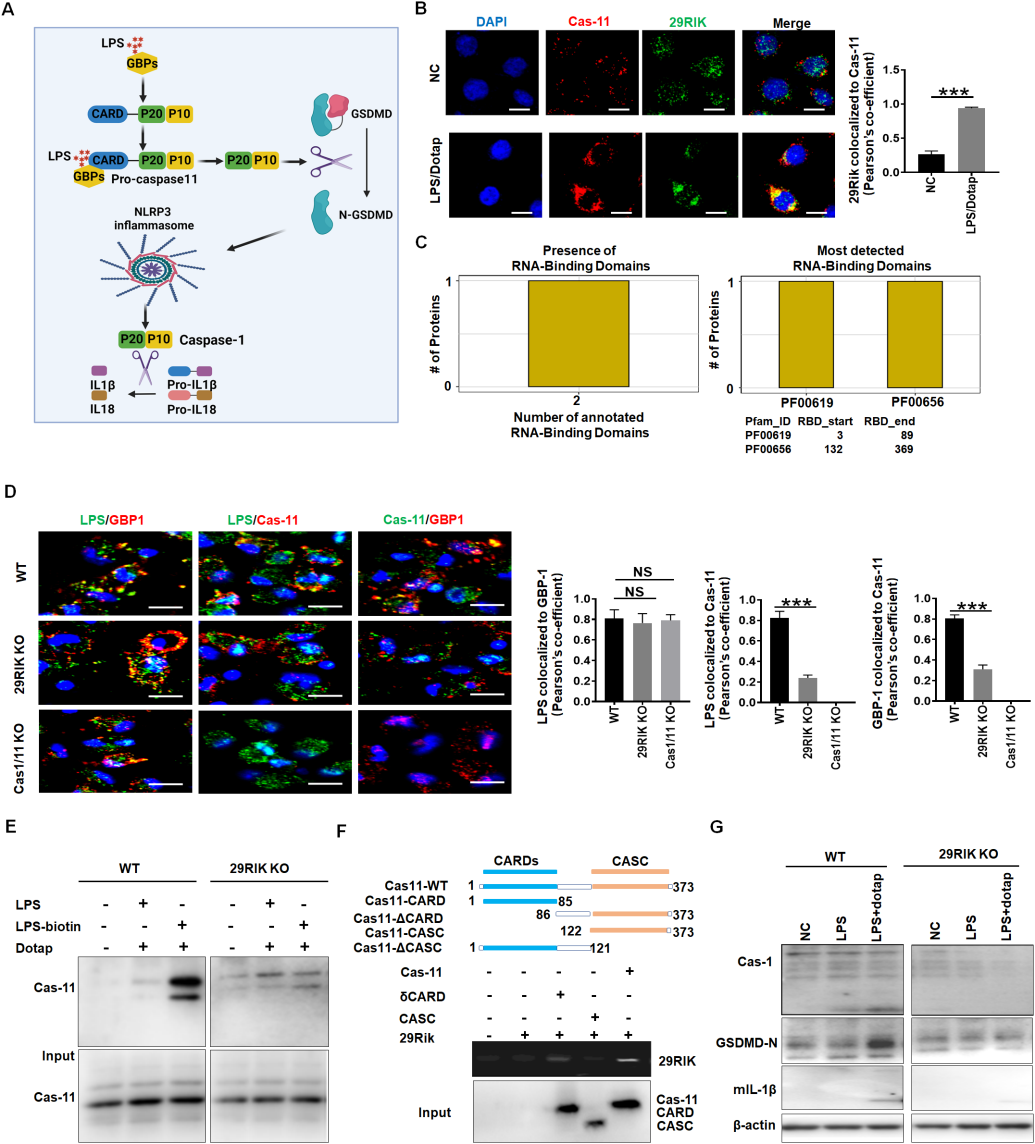
*LncRNA29RIK* (29RIK) promote the binding of LPS with caspase-11. **(A)** Schematic illustration showing the LPS mediated mIL-1β and pyroptosis. **(B)** Immunostaining of caspase-11 and *LncRNA29RIK* in the macrophages after exposure to LPS/dotap. Pearson’s correlation coefficient indicating co-localization of caspase-11 (cas-11) and *LncRNA29RIK (29rik).* DAPI, nuclear staining; NC, control; **(C)** Predicting binding of mouse *lncRNA29RIK* with caspase-11. The prediction results showed that there were two domains binding to *lncRNA29RIK* in caspase-11 protein, and the amino acid start and end site of the domain was given. **(D)** Immunostaining of LPS, GBP1, and caspase-11 (Cas-11) in the WT, *LncRNA29RIK* (29Rik) KO, and caspase-1/caspase-11 (Casp1/11) KO macrophages. Pearson’s correlation coefficient indicating co-localization of LPS and GBP1, LPS/Casp11, and Cas-11/GBP in 29 RIK KO and Cas-1/11 KO macrophages; WT, control. **(E)** Immunoblotting of caspase-11 (Cas-11) in the biotin-labeled LPS pull-down lysis of the WT and *lncRNA29RIK* KO microphages. **(F)** RIP of V5-tagged caspase-11 derivatives and *lncRNA29RIK* cotransfected HEK293T cells. RIP was performed using anti-V5. Caspase-11 and its derivatives were cloned into pcDNA3.1/V5 to generate V5-tagged caspase-11 derivatives and then individually transfected into HEK293T cells. **(G)** Immunoblotting of caspase-1 (Cas-1), cleaved GSDMD (GSDMD-N), and mIL-1β in the WT and *lncRNA29RIK* KO (29RIK KO) macrophages.

We next investigated the mechanism(s) by which *mlncRNA29RIK* regulated the production of mIL-1β and macrophage pyroptosis. Caspase-11, as a non-canonical inflammasome, was activated by oligomerization of the LPS/caspase-11 complexes (29). Activation of caspase-11 could induce proteolysis of the full length of GSDMD to generate the N-GSDMD pore-forming domain, which migrates to cell membranes to produce the N-GSDMD-mediated membrane pores to facilitate potassium iron efflux. Notably, NLRP3 canonical inflammasomes can be activated via potassium ion efflux, which subsequently induces proteolytic activation of caspase-1 to cause release of mIL-1 β (30) (**Figure 4A**). Thus, we first analyzed the roles of *lncRNA29RIK* in the oligomerization of the LPS/caspase-11 complexes. Data showed that *lncRNA29RIK* could gather together with LPS/caspase-11 upon exposure to LPS/dotap (**Figure 4B**), implying that *lncRNA29RIK* exerts its role through caspase-11. LncRNAs can serve as scaffolds in the cytoplasm to nucleate complex networks of proteins functioning in tightly regulated signaling transduction programs (31–33). Bioinformatics analyses showed that this *lncRNA29RIK* could potentially bind with caspase-11 (**Figure 4C**). Indeed, immunofluorescence showed that *lncRNA29RIK* could promote the oligomerization of caspase-11 (**Figure 4D**, **Supplementary Figure S4**). However, *lncRNA29RIK* was not involved in the binding of LPS and GBP, which could promote the recruitment of caspase-4/11 to trigger its activation (11). There was no difference in the binding of LPS and GBP in WT, *lncRNA29RIK*, and caspase-1/11 KO macrophages, whereas markedly less assemble of LPS and caspase-11, and caspase-11 and GBP1 were observed in *lncRNA29RIK* KO and *caspase 1/11* KO macrophages as compared to WT macrophages (**Figure 4D**). LPS pull-down experiment also exhibited that binding of LPS with caspase-11 was dependent on *lncRNA29RIK* (**Figure 4E**). Interestingly, RNA immunoprecipitation (RIP) showed the direct binding of *lncRNA29RIK* with CARD domain in caspase-11 (**Figure 4F**). The interaction of *lncRNA29RIK* and caspase-11 promoted the degradation of caspase-1 and GSDMD and production of mIL-1 β in WT but not *lncRNA29RIK* KO macrophages upon exposure to LPS/dotap (**Figure 4G**). In addition, we also analyzed the coding potential of *lncRNA29RIK*. The phyloCSF showed that the *lncRNA29RIK* did not have a coding potential (**Supplementary Figure S5**). Coding Potential Calculator 2 (CPC2, http://cpc2.gao-lab.org/) analyses also revealed that this *lncRNA29RIK* did also not have the potential to encode peptides (**Supplementary Figure S5**). Thus, our results suggest that *lncRNA29RIK* can bind with caspase-11 to promote the oligomerization of LPS-mediated caspase-11, which can cause production of mIL-1β and pyroptosis of macrophages.

### 3.5 *LncRNA29RIK* has similar function to caspase-11

**Figure 5:**
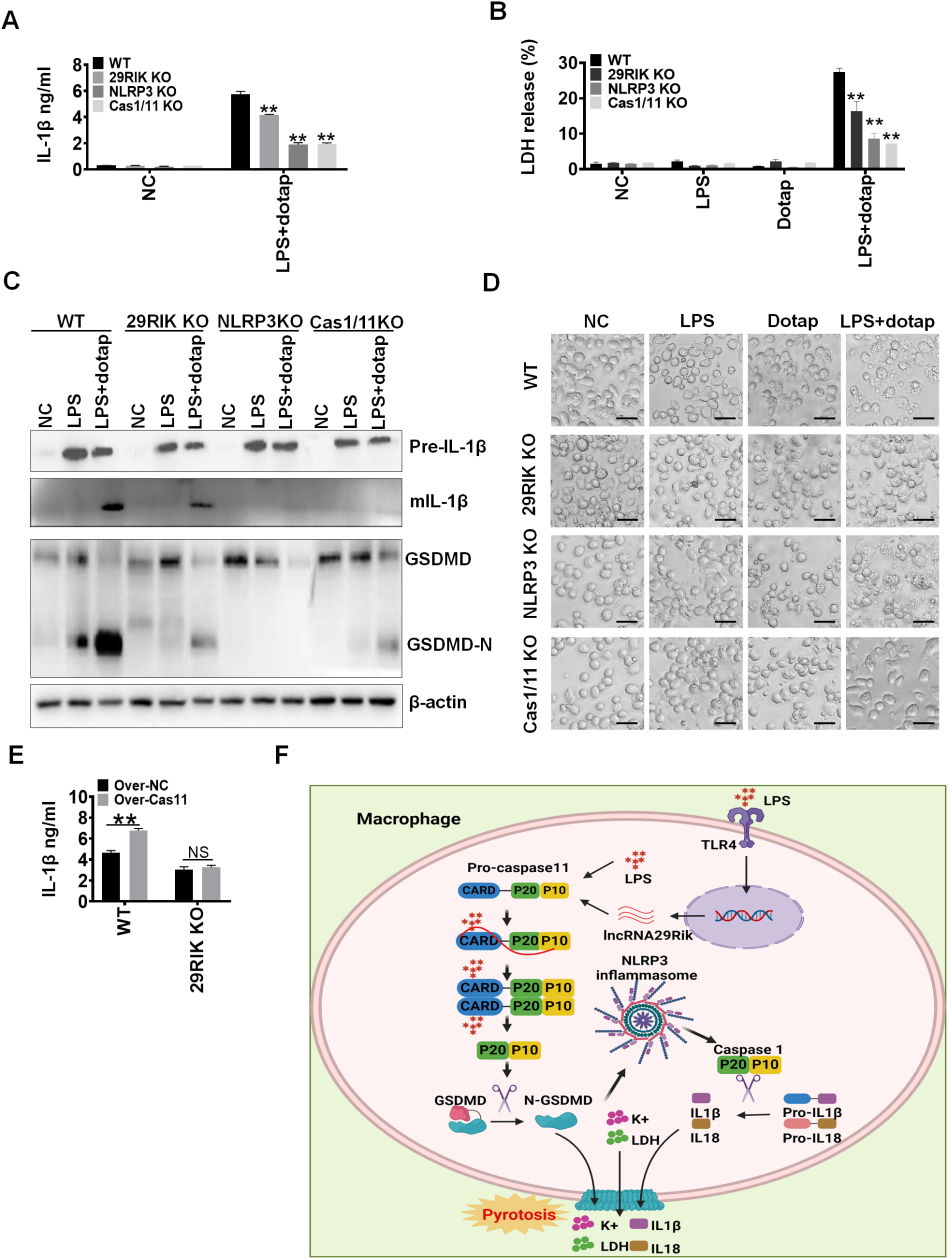
There has a similar function among *lncRNA29RIK* (29RIK) KO, caspase-1/11 and NLRC3 KO macrophages. **(A)** ELISA of IL-1β in the supernatants of *lncRNA29RIK* KO, caspase-1/11, and NLRC3 KO macrophages after exposure to LPS/dotap. NC, control. **(B)** Analyses of LDH in the supernatants of *lncRNA29RIK* KO, caspase-1/11, and NLRC3 KO macrophages after exposure to LPS/dotap. NC, control. **(C)** Immunoblotting of pro-IL-1β, IL-1β, GSDMD, and cleaved GSDMD (GSDMD-N) in the *lncRNA29RIK* KO, caspase-1/11, and NLRC3 KO macrophages after exposure to LPA/dotap. **(D)** Pyroptosis of *lncRNA29RIK* KO, caspase-1/11, and NLRC3 KO macrophages after exposure to LPS/dotap. NC, control. **(E)** ELISA of IL-1β in the supernatants of caspase-11 overexpressed *lncRNA29RIK* KO macrophages after exposure to LPS/dotap; WT, control macrophages. **(F)** Schematic illustration showing a mechanism for *lncRNA29RIK* to induce production of IL-1β and pyroptosis of macrophages. Student’s *t*-test; *p < 0.05, **p < 0.01, ***p < 0.001; NS, not significant.

LPS-mediated mIL-1β and pyroptosis of macrophages was through multiple components such as caspase-11 and NLRP3 (30). To further understand how *lncRNA29RIK* mediated mIL-1β and macrophage pyroptosis, we used KO (KO) mice to compare the basic function of *lncRNA29RIK*, caspase-11, and NLRP3 in inducing mIL-1 β production and macrophage pyroptosis. Data showed that not only *lncRNA29RIK* KO but also *caspase-11* KO and *NLRP3* KO macrophages had reduced mIL-1β production and decreased pyroptosis of macrophages (**Figures 5A, B**). There were no or weak responses to LPS/dotap stimulation in the production of mIL-1β and cleaved GSDMD in these *lncRNA29RIK* KO, *caspase-11* KO, and *NLRP3* KO macrophages upon exposure to LPS/dotap (**Figure 5C**). Much fewer pyroptosis cells in these KO mice were also observed (**Figure 5D**). Notably, overexpression of caspase-11 did not rescue IL-1β production in *lncRNA29RIK* KO macrophages (**Figure 5E**). Thus, there have been similar function in *lncRNA29RIK* KO, *caspase-11* KO, and *NLRP3* KO macrophages in inducing the production of mIL-1β and pyroptosis of macrophages, supporting that the functions of *lncRNA29RIK* are dependent on caspase-11 and NLRP3 (**Figures 5E, F**).

### 3.6 *LncRNA29RIK* promotes sensitivity to LPS-mediated inflammation

**Figure 6:**
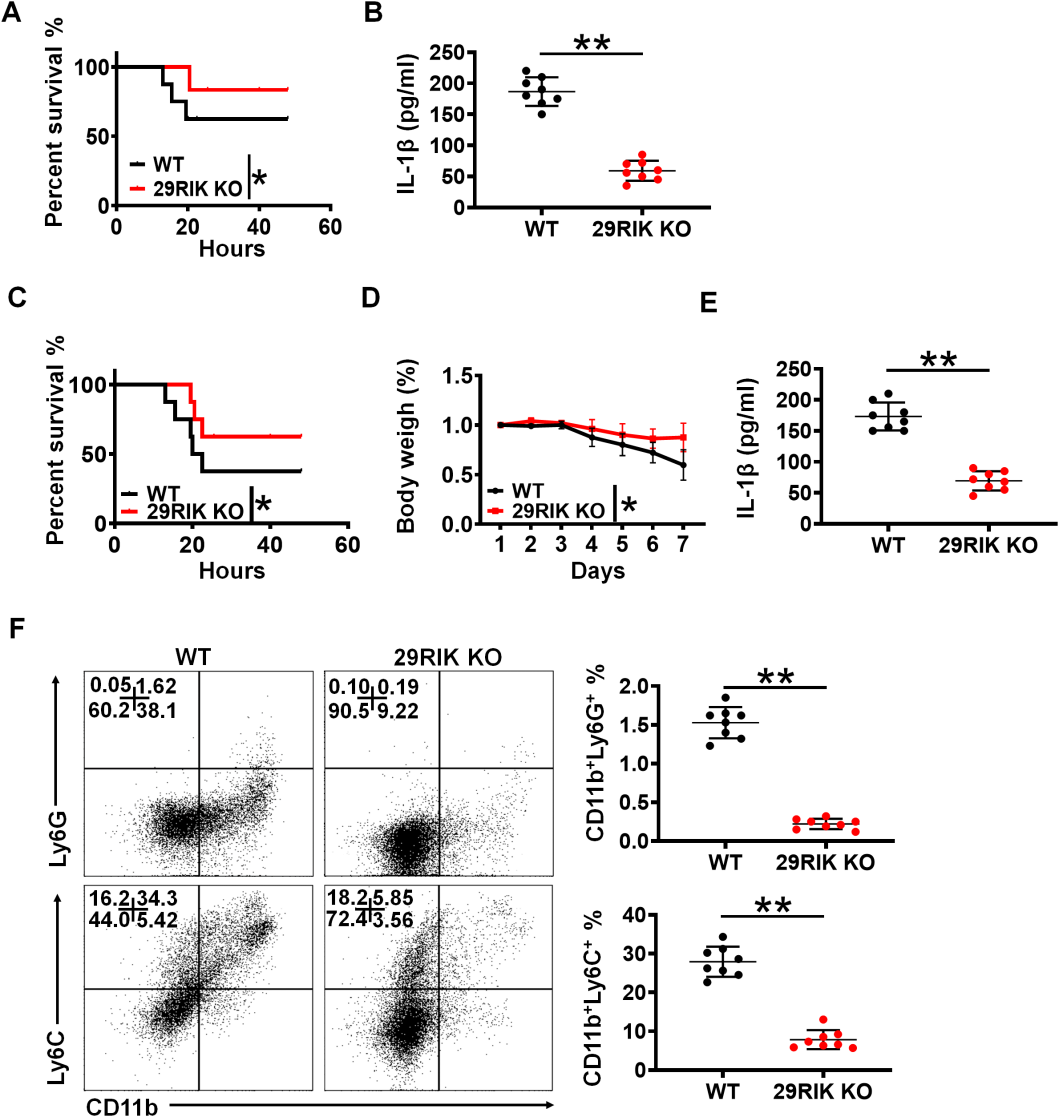
*lncRNA29RIK* (29RIK) promotes LPS-mediated inflammation *in vivo*. **(A)** Survival rate of WT and *lncRNA29RIK* KO mice with or without intraperitoneal injection LPS (52 mg/kg) (n=8). **(B)** ELISA of IL-1β in the sera of WT and *lncRNA29RIK* KO mice with or without intraperitoneal injection LPS (52 mg/kg) (n=8). **(C)** Survival rate and body weight of WT and *lncRNA29RIK* KO mice with or without *S. typhimurium* infection (200 CFUs/mouse, n=8). **(D)** Body weight of WT and *lncRNA29RIK* KO mice with or without *S. typhimurium* infection (200 CFUs/mouse, n=8). **(E)** ELISA of IL-1β in the sera of WT and *lncRNA29RIK* KO mice with or without *S. typhimurium* infection (200 CFUs/mouse, n=8). **(F)** Follow cytometry of CD11B^+^Ly6G^+^ and CD11b^+^ Ly6C^+^ cells in the colon tissues of WT and *lncRNA29RIK* KO mice with or without *S. typhimurium* infection (200 CFUs/mouse, n=8). One-way ANOVA Bonferroni’s multiple comparison test in part **(D)**; Wilcoxon’s test in parts **(A, C)**; two-sided Student’s *t*-test in **(B, E, F)** **p < 0.01, ***p < 0.001; NS, not significant.

We next determined the role of macrophage *mlncRNA29RIK* in LPS or Gram-negative bacteria-mediated inflammation. LPS toxic analyses showed that *mlncRNA29RIK* KO mice had higher survival rate than WT mice (**Figure 6A**). In LPS toxic experiments, these *lncRNA29RIK* KO mice had lower levels of mIL-1β in sera (**Figure 6B**).

To further determine the function of *mlncRNA29RIK* in the macrophages, we also employed *S. typhimurium* infection models. This *mlncRNA29RIK* KO and WT mice were individually infused with *S. typhimurium* (200 CFUs/mouse). As compared to *mlncRNA29RIK* KO mice, WT mice had markedly reduced body weight and survival rate and increased mIL-1β in the sera (**Figures 6C–E**). Meanwhile, there were also less inflammatory immune cells in the colon tissues of *mlncRNA29RIK* KO mice than WT mice (**Figure 6F**). Taken together, all of these suggest that *mlncRNA29RIK* in the macrophages plays a critical role in LPS-mediated inflammation.

### 3.7 Similar functions in the *lncRNA29RIK* between mice and human

**Figure 7:**
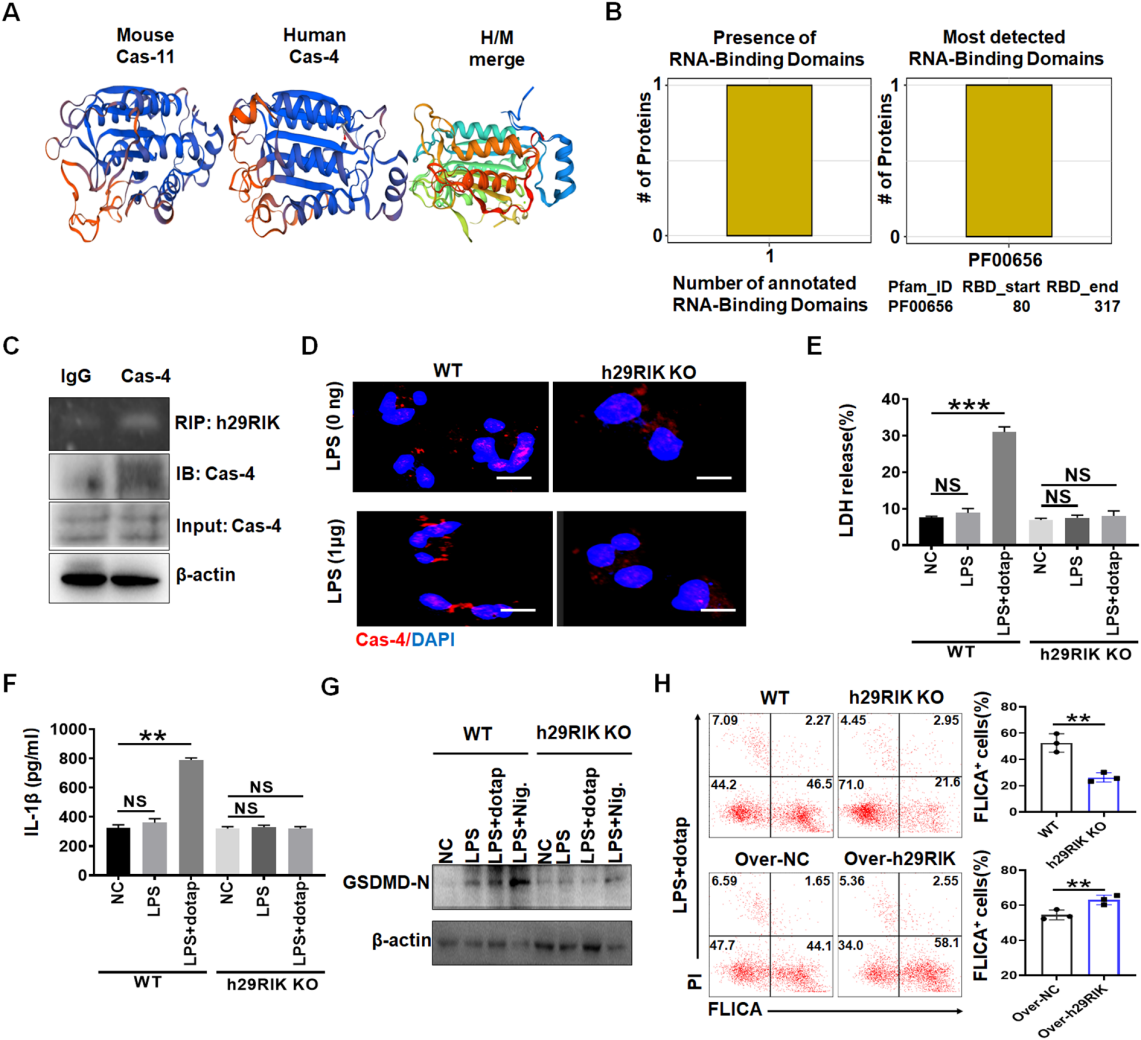
Human *lncRNA29RIK* (29RIK) have similar function with mice. **(A)** Schematic illustration showing mouse caspase-11 and human caspase-4 structures. **(B)** Predicting binding of human *lncRNA29RIK* with caspase-4. The prediction results showed that there were one domain binding to human *lncRNA29RIK* in caspase-4 protein, and the amino acid start and end site of the domain was given. **(C)** RIP of V5-tagged caspase-4 and human *lncRNA29RIK* co-transfected HEK293T cells. RIP was performed using anti-V5. % input of human *lncRNA29RIK* was analyzed. Caspase-4 were cloned into pcDNA3.1/V5 to generate V5-tagged caspase-4, and then individually transfected into HEK293T cells. **(D)** Immunostaining of caspase-4 (Cas-4) in human-monocyte-derived macrophages after exposure to LPS. **(E)** Analyses of LDH in the supernatants of human *lncRNA29RIK* KO macrophages (h29RIK KO) after exposure to LPS/dotap. NC, control. **(F)** ELISA of IL-1β in the supernatants of human *lncRNA29 RIK* KO macrophages (h29RIK KO) after exposure to LPS/dotap. NC, control. **(G)** Immunoblotting of cleaved GSDMD (GSDMD-N) in the human *lncRNA29RIK* KO macrophages (h29RIK KO) after exposure to LPS/dotap or LPS+ Nigericin (LPS+Nig). **(H)** Flow cytometry of pyroptosis cells in *lncRNA29RIK* overexpressed macrophages after staining using FLICA upon exposure to LPS+Dotap. Cont, control lncRNA; WT, control macrophages. NC, control. Two-sided Student’s *t*-test in parts **(A–G)**. **p < 0.01, ***p < 0.001; NS, not significant.

Finally, we investigated whether there existed a similar function in the *lncRNA29RIK* between human and mice. Bioinformatics analyses showed that *lncRNA29RIK* was highly conserved between mouse and human (https://blast.ncbi.nlm.nih.gov/Blast.cgi or DNAMAN software) (**Supplementary Figure S1**). Caspase-11 and caspase-4 also had a similar 3D structure (**Figure 7A**). There also existed multiple potential binding sites on the caspase-4 with human *lncRNA29RIK* (*hulncRNA29RIK*) (**Figure 7B**). Indeed, RIP analyses showed the binding of *lncRNA29RIK* with caspase-4 (**Figure 7C**). Immunofluorescence also showed increased oligomerization of the LPS/caspase-4 complexes after exposure to LPS/dotap (**Figure 7D**). To further determine function(s) of *hulncRNA29RIK* in the human macrophages, we generated *hulncRNA29RIK* KO (KO) THP1 cells using CRISPR/cas9 technique. THP1, which can be induced into macrophages, is often used as a macrophage model of macrophages (34, 35). Results showed that *hulncRNA29RIK* also had similar function with mice. There was no difference in the pyroptosis of *hulncRNA29RIK* KO THP1 cells with and without exposure to caspase-4 ligand LPS/dotap, whereas marked differences can be observed in WT THP1 cells (**Figure 7E**). There were no changes in the mIL-1β production in *hulncRNA29RIK* KO THP1-derived macrophages with and without exposure to LPS/dotap, whereas marked differences could be observed in WT macrophages (**Figure 7F**). Cleaved GSDMD also increased in WT THP1 cells but not in *hulncRNA29RIK* KO THP1 cells upon exposure to LPS/dotap (**Figure 7G**).

*LncRNA29RIK* overexpression also enhanced pyroptosis in human primary macrophages (**Figure 7H**). Thus, *hulncRNA29RIK-*mediated mIL-1β maturation and pyroptosis is via binding with caspase-4. All of these suggest that there exists a similar function in the *lncRNA29RIK* between human and mice.

## 4 Discussion

We here demonstrate that *lncRNA29RIK* can bind with caspase-11/4 to promote the intracellular LPS-mediated inflammation. This *lncRNA29RIK* can act as scaffolds to promote the oligomerization of LPS-mediated caspase-4/11, which can cause inflammatory cytokine IL-1β maturation and cellular pyroptosis in the macrophages. These results offer a potential target for controlling LPS-associated diseases.

Previous studies showed that caspase-4/11 activation by LPS or Gram-negative bacteria requires the expression of interferon (IFN)-inducible guanosine triphosphate (GTP)ases, such as guanylate-binding proteins (GBPs) and/or immunity-related GTPases (IRGs) (7–10). A complex of GBP and LPS could promote the recruitment of caspase-4/11 and subsequently transfer LPS onto caspase-4/11 to trigger its activation (11). It is unclear how LPS is transferred to caspase-4/11 via GTPase. Here, we demonstrate that *lncRNA29RIK* participates in this process by binding to caspase-4/11 (human/mouse), thereby promoting the activation of caspase-4/11.

We demonstrate that *lncRNA29RIK* can act as scaffolds to promote the activation of LPS-mediated caspase-4/11. *LncRNAs* can associate with RNA-binding proteins (RBPs) to form lncRNA–protein complexes, which are involved in a wide range of biological processes (36). For example, LncRNA HULC promoted phosphorylation through directly binding to glycolytic enzymes, lactate dehydrogenase A (LDHA), and pyruvate kinase M2 (PKM2) to (37); and LINK-AlncRNA enhanced the recruitment of BRK to the EGFR: GPNMB complex and BRK kinase activation (32); LNCRNAAK023948 is necessary for the interaction between DHX9 and p85, hence the p85 stability and promote AKT activity (38). Others also demonstrated that cytoplasmic lncRNAs could participate in regulating protein stability and modification (39, 40).

We found that *LncRNA29RIK* in macrophages promotes *LPS-*mediated sensitivity to obesity. Most macrophages in adipose tissues of obesity are M1 (inflammatory) macrophages, whereas M2 (immunosuppressive) macrophages exist in adipose tissues of thin individual. Inflammatory macrophages that accumulate in adipose tissues of obesity play a critical role in the occurrence and development of obesity. These macrophages have been shown to increase the expression of inflammatory cytokines, thereby causing chronic inflammation (41). LPS derived from gut microbiota is a potential factor for inducing inflammatory responses in the macrophages of adipose tissue (42). In addition, LPS-mediated caspase-4/11 signaling also appears in sepsis (43), diabetes (44), atherosclerosis (45), and Alzheimer’s disease (46) in acute and chronic inflammatory conditions. Thus, our data also offer a potential target for controlling these diseases. Currently, a variety of targeted treatments for lncRNAs have been developed, such as anti‐sense oligonucleotides, liposome/nanoparticle‐delivered siRNAs, and small‐molecule inhibitors of lncRNAs (47).

Notably, the activation of AIM2 in response to LPS is also an interesting and novel result of the current study (48, 49). Whether AIM2 also plays a role in *LncRNA29RIK-*mediated sensitivity to obesity needs to be further investigated.

## Data Availability

The datasets presented in this study can be found in online repositories. The names of the repository/repositories and accession number(s) can be found in the article/**Supplementary Material**.
