# Supplementary material for: LncRNA29RIK in macrophages promotes LPS-mediated sensitivity to obesity
Source: Front Immunol. 2025 Apr 28;16:1574507. doi: 10.3389/fimmu.2025.1574507 (PMC12066258; doi:10.3389/fimmu.2025.1574507)
Supplement: Supplementary file 2 [file Table1.docx]

***LncRNA29RIK* in macrophages determines *LPS* mediated sensitive to obesity**

Rong Wang, Yunhuan Gao, Yuan Zhang, and Rongcun Yang ^1,2,3^

^1^ Department of Immunology, Nankai University School of Medicine; Nankai University, Tianjin 300071, China;

^2^Translational Medicine Institute, Affiliated Tianjin Union Medical Center of Nankai University, Tianjin 300071, China;

^3^State Key Laboratory of Medicinal Chemical Biology, Nankai University, Tianjin 300071, China;


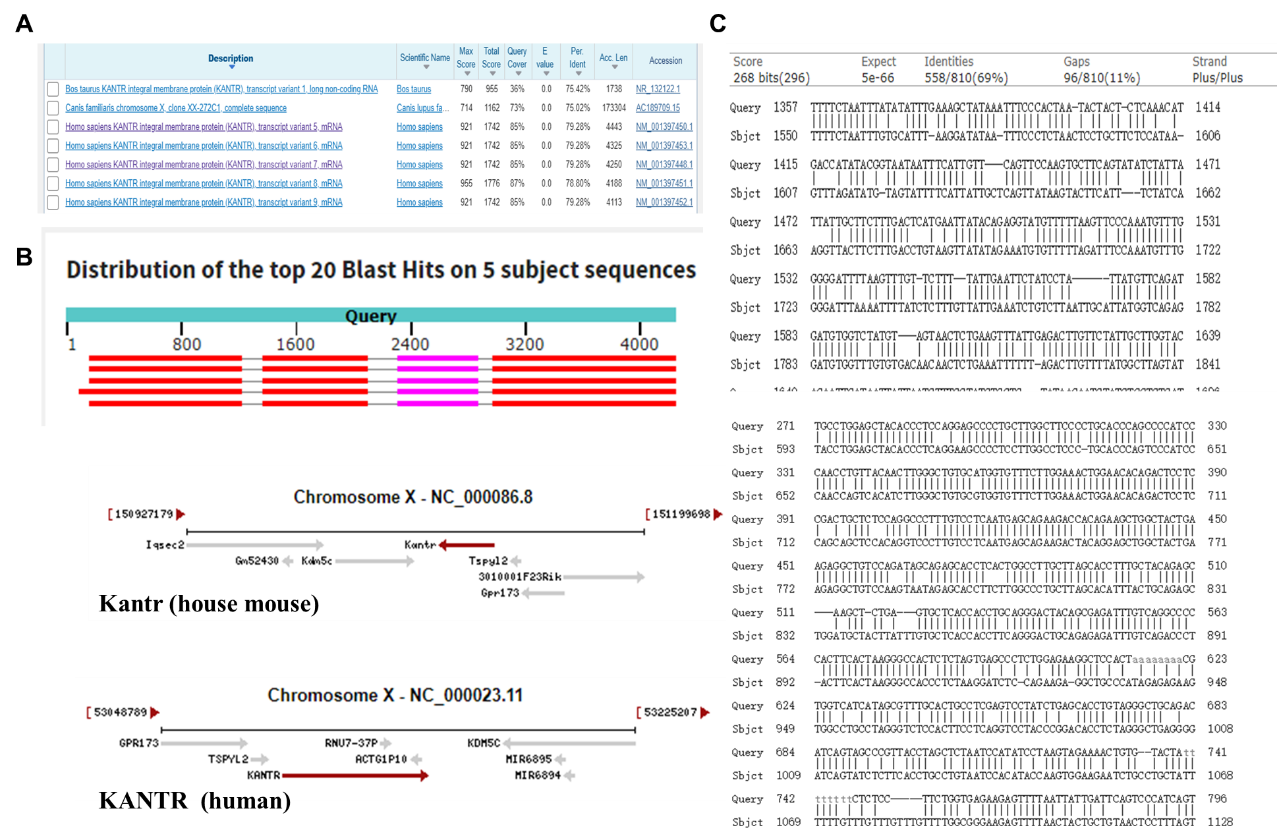


**Figure S1.** The location and structure of *lncRNA29RIK*. (A and B) The location and structure of *lncRNA29RIK* in chromosome. (C) Compared of human and mouse *lncRNA29RIK* sequences.


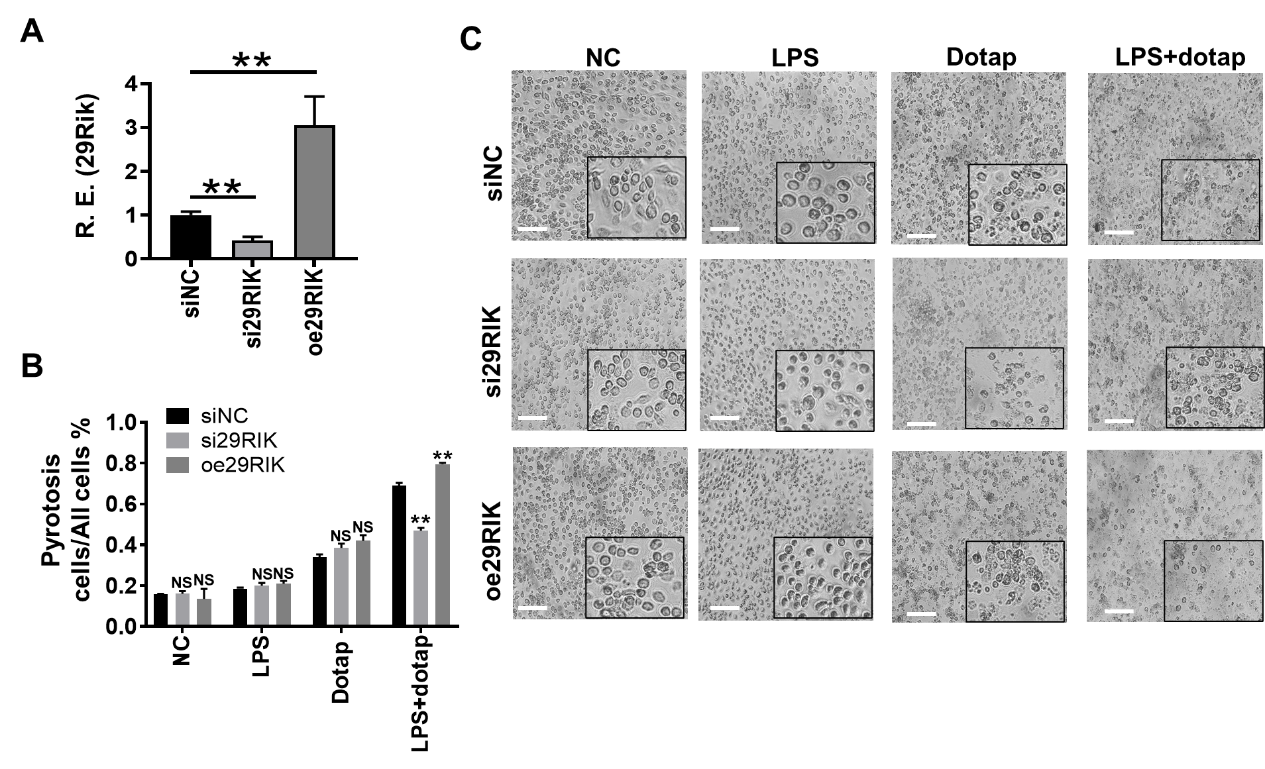


**Figure S2.** *LncRNA29RIK* induces pyroptosis of macrophages after exposure to LPS/dotap. (A) qRT-PCR of *LncRNA29RIK in* lncRNA29RIK siRNA or exogenous lncRNA29RIK transfected macrophages; (B) Analyses of pyroptosis cells in macrophages after exposure to LPS/dotap; (C) Morphology of the macrophages after exposure to LPS/dotap. Student’s *t*-test, mean ±SD. ***P* < 0.01; Ns, no significance; Data are a representative of three independent experiments.


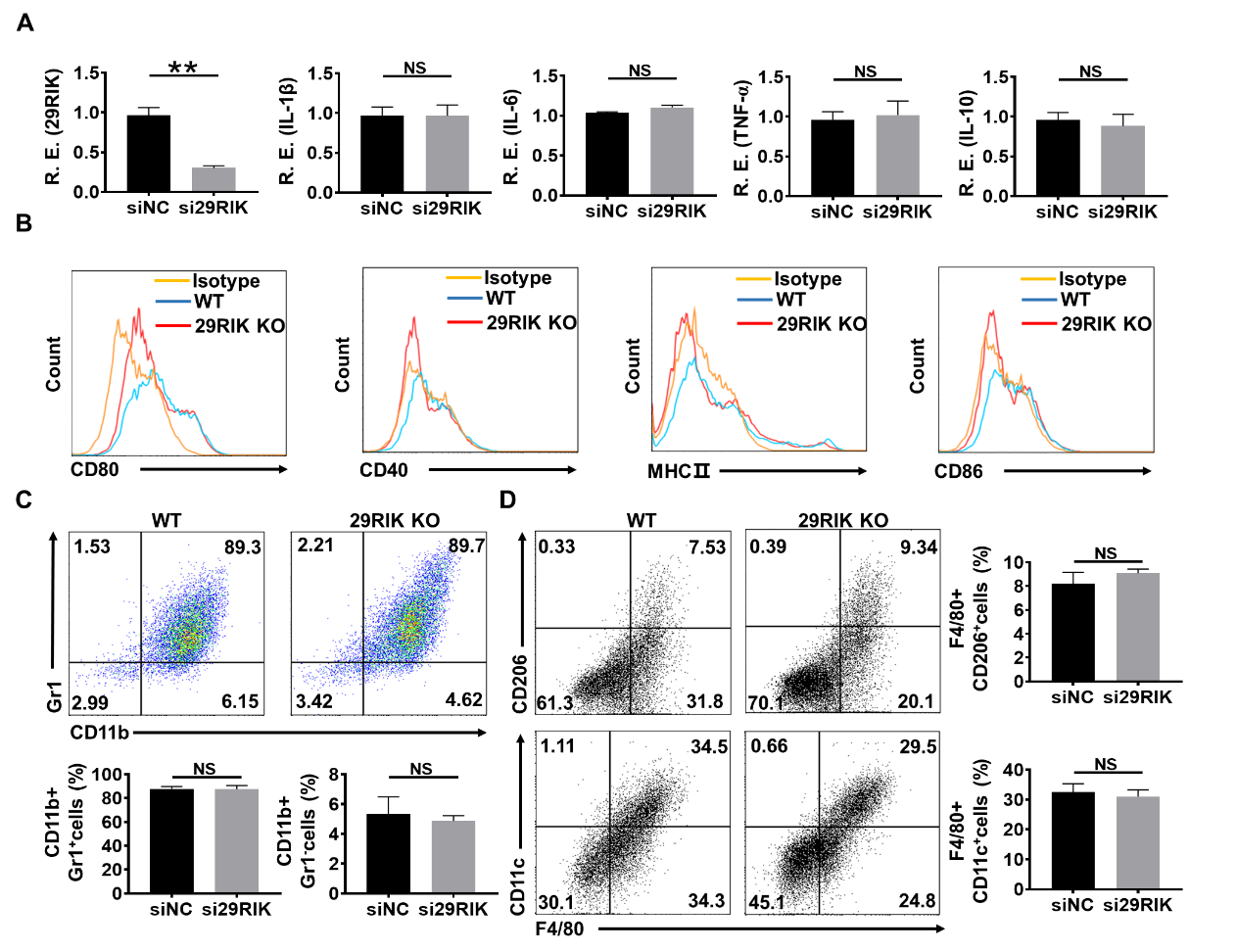


**Figure S3.** *LncRNA29RIK* does not affect the gene expression and differentiation of immune cells. (A) qRT-PCR in the *LncRNA29RIK* KO macrophage. R. E., relative expression. (B) Flow cytometry of CD80, CD40, MHC II and CD86 in the *LncRNA29RIK* KO macrophage. (C) Flow cytometry of CD11b^+^Gr-1^+^ in the *LncRNA29RIK* KO bone marrow cells. (D) Flow cytometry of F4/80^+^CD11C^+^ and F4/80^+^CD206^+^ cells in the *LncRNA29RIK* KO bone marrow cells. Student’s *t*-test, mean ±SD. ***P* < 0.01; Ns, no significance; Data are a representative of three independent experiments.


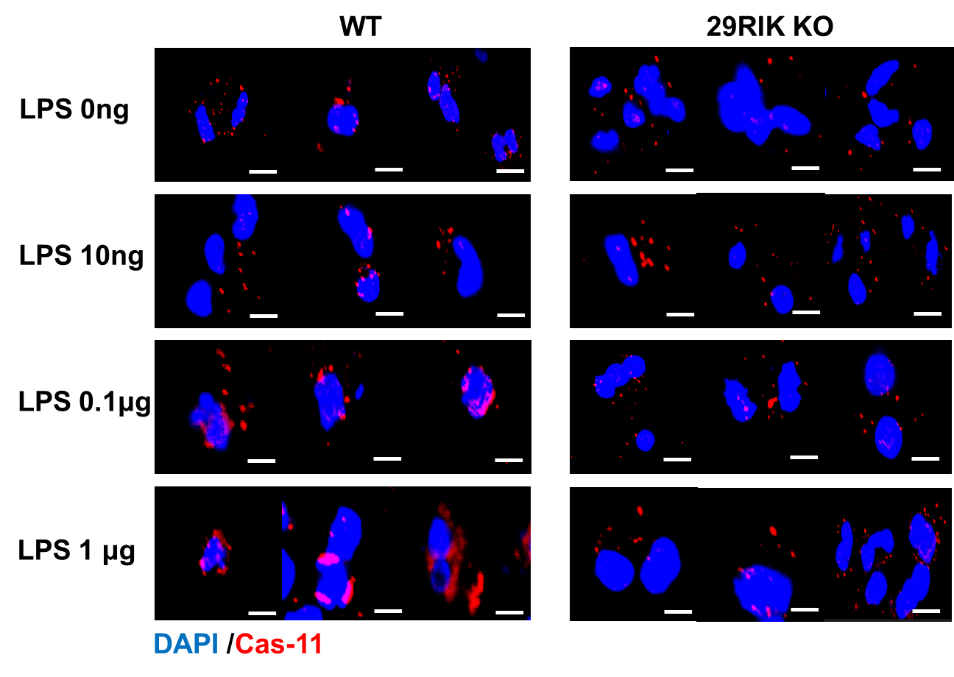


**Figure S4.** Immunostaining of capsase-11 in *lncRNA29RIK* KO macrophages after exposure to different concentration of LPS.


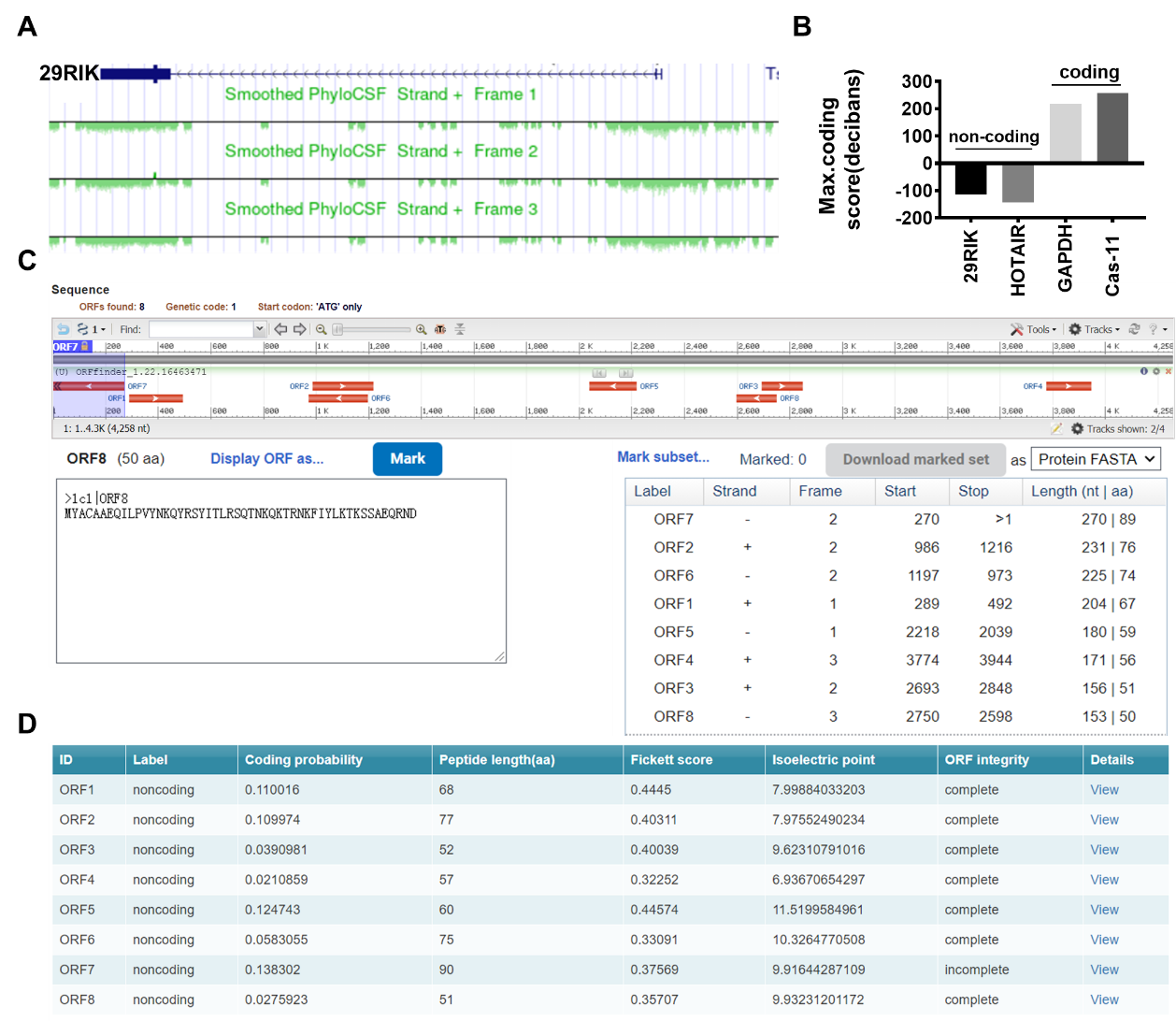


**Figure S5.** Analyses of coding potential of *lncRNA29RIK.* (A and B) Coding potency of *lncRNA29Rik* sequence was analyzed using PhyloCSF. Hotair, a control non-coding gene. GAPDH and Caspase 11, control coding genes. Scores above 0 suggested there had a coding potential, whereas scores below 0 represented no coding potential. (C) Open reading frame on *lncRNA29RIK* by ORF finder (<https://www.ncbi.nlm.nih.gov/orffinder/>). (D) Encoding capability of *lncRNA29RIK* and open reading frame by CPC2 (Coding Potential Calculator2, <http://cpc2.gao-lab.org/>).

**Table S1．Reagents and oligoes used in this study.**

| REAGENT or RESOURCE | SOURCE | IDENTIFIER |
| --- | --- | --- |
| Antibodies | | |
| β-Actin Antibody | Santa Cruz | Cat:sc-47778 RRID: AB_626632 |
| Anti-Caspase 4 Antibody | Thermo Fisher Scientific | Cat: PA5-21286 RRID: AB_11153341 |
| Anti-caspase-4 (4B9) Antibody | Santa Cruz Biotechnology | Cat: sc-56056, RRID: AB_781828 |
| V5 Tag Monoclonal Antibody | Thermo Fisher Scientific | Cat:MA5-15253 RRID: AB_10977225 |
| Anti-Caspase11 antibody | Cell Signaling Technology | Cat: 14340 RRID: AB_2728693 |
| Anti -IL1β antibody | Proteintech | Cat: 16806-1-AP RRID: AB_10646432 |
| Anti- Cleaved Caspase-1 antibody | Cell Signaling Technology | Cat：67314 RRID: AB_2714037 |
| F4/80 (6A545) antibody | Santa Cruz | Cat:sc-71085 RRID: AB_1122717 |
| APC-CD45 30-F11 | Biolegend | Cat:103112 RRID: AB_312977 |
| FITC-CD11b M1/70 | eBioscience | Cat:11-0112-86 RRID: AB_464937 |
| PE-Ly6G 1A8 | Biolegend | Cat: 127607 RRID: AB_1186104 |
| PE-F4/80 | Biolegend | Cat: 157303 RRID: AB_2832547 |
| PE Gr1 | Biolegend | Cat: 108407 RRID: AB_313372 |
| FITC CD11c | Biolegend | Cat: 117305 RRID: AB_313774 |
| FITC CD206 | Biolegend | Cat: 162507 RRID: AB_3097515 |
| PE-Ly6C | Biolegend | Cat: 128007 RRID: AB_1186132 |
| PE CD86 | Biolegend | Cat: 159203 RRID: AB_2832567 |
| FITC CD40 | Biolegend | Cat: 124608 RRID: AB_1134090 |
| FITC CD80 | Biolegend | Cat: 104705 RRID: AB_313126 |
| FITC MHC Class II (I-A/I-E) | eBioscience | Cat: 11-5321-82 RRID: AB_465232 |
| Bacterial Strains | | |
| S. Typhimurium | ATCC | Cat: 14028 |
| Chemicals, Peptides, and Recombinant Proteins | | |
| Recombinant Murine GM-CSF | PeproTech | Cat:315-03 |
| Recombinant Murine M-CSF | PeproTech | Cat:315-02 |
| Recombinant Human M-CSF | PeproTech | Cat: 300-25 |
| Recombinant Human IFN-γ | PeproTech | Cat: 300-02 |
| Recombinant Murine IFN-γ | PeproTech | Cat: 315-05 |
| HiPerFect Transfection Reagent | QIAGEN | Cat:301705 |
| DOTAP chloride | Selleck | Cat: S6908 |
| LPS (0111:B4) | Sigma | Cat: L2630 |
| Nigericin | MedChemExpress | Cat: 28380-24-7 |
| Flagellin | AdipoGen Life Sciences | Cat: AG-40B-0095 |
| Lipofectamine™ 3000 Transfection Reagent | Thermo Fisher Scientific | Cat:11668027 |
| Trizol | Life technologies | Cat:15596018 |
| Experimental Models: Cell Lines | | |
| HEK 293T | ATCC | N/A |
| THP-1 | ATCC | N/A |
| Oligonucleotides for clone genes | | |
| Murine Caspase 11 FW | BGI | 5’- ATGGCTGAAAACAAACACCC -3’ |
| Murine Caspase 11 REV | BGI | 5’- GTTGCCAGGAAAGAGGTAGA-3’ |
| Human Caspase 4 FW | BGI | 5’- ATGGCAGACTCTATGCAAGA-3’ |
| Human Caspase 4 REV | BGI | 5’- ATTGCCAGGAAAGAGGTAGAA-3’ |
| Murine Caspase11 (CARD) FW | BGI | 5’- ATGGCTGAAAACAAACACCC -3’ |
| Murine Caspase11 (CARD) REV | BGI | 5’-GTCCACACTGAAGAATGTCT-3’ |
| Murine Caspase11(LS) FW | BGI | 5’-ATGCCAGGCAGCCACCATGGT-3’ |
| Murine Caspase11(LS) REV | BGI | 5’- CTCTCTGATCCACATTTCTC-3’ |
| Murine Caspase11(SS) FW | BGI | 5’-ATGTCTTCAAAACCCCAGTTG-3’ |
| Murine Caspase11(SS) REV | BGI | 5’- GTTGCCAGGAAAGAGGTAGA-3’ |
| Oligonucleotides for qRT-PCR | | |
| Murine GAPDH FW | BGI | 5’-TCAACGGCACAGTCAAGG-3’ |
| Murine GAPDH REV | BGI | 5’-TACTCAGCACCGGCCTCA-3’ |
| Murine lncRNA29Rik FW | BGI | 5’- GACTAGAGGTCCGTGGGTGG -3’ |
| Murine lncRNA29Rik REV | BGI | 5’- AATGGTGGATGTTCTGGGTA -3’ |
| Murine Caspase11 FW | BGI | 5’- GAGAAATGTGGATCAGAGAG-3’ |
| Murine Caspase11 REV | BGI | 5’- GATGTGGGGTTGTAGAGTAG-3’ |
| Murine IL1βFW | BGI | 5’-TCGCAGCAGCACATCAACAAG-3’ |
| Murine IL1βREV | BGI | 5’-GAAGGTCCACGGGAAAGACAC-3’ |
| Murine TNFa FW | BGI | 5’- GTTCATCCATTCTCTACCCA -3’ |
| Murine TNFa REV | BGI | 5’- GAGCCATAATCCCCTTTCTA -3’ |
| Murine MCP-1(Ccl2) FW | BGI | 5’- ACCTTTTCCACAACCACCTC-3’ |
| MurineMCP-1(Ccl2) REV | BGI | 5’- GGATCCACACCTTGCATTTA-3’ |
| Murine IL6 FW | BGI | 5’-AGACTTCCATCCAGTTGCCT-3’ |
| Murine IL6 REV | BGI | 5’-TCTCATTTCCACGATTTCCC-3’ |
| Human GAPDH FW | BGI | 5’- TCAAGAAGGTGGTGAAGCAGG-3’ |
| Human GAPDH REV | BGI | 5’- AGCGTCAAAGGTGGAGGAGTG-3’ |
| Human lncRNA29Rik FW | BGI | 5’-ACTAAAGGTTGAAATCAGAGAC-3’ |
| Human lncRNA29Rik REV | BGI | 5’- CAACAGGGCGAGACTACGTC -3’ |
| Human Caspase4 FW | BGI | 5’- AGAGGTGCAAACCGTGGGGAA -3’ |
| Human Caspase4 REV | BGI | 5’- CGTTGTGTGGCGTTGAAGAGC -3’ |
| Human IL1β FW | BGI | 5’- GGCAATGAGGATGACTTGTTC-3’ |
| Human IL1β REV | BGI | 5’- TGCTGTAGTGGTGGTCGGAGA-3’ |
| Probes used in the RNA-FISH | | |
| Murine lncRNA29Rik -FAM | BGI | 5’-FAM- CTTCACGCAATGCTCCACCGA -3’ |
| NC-FAM | BGI | 5’-FAM-CGGGAGCCTAGGAAGTGCATCTTTC-3’ |
| siRNAs used in this study | | |
| Murine lncRNA29Rik | Ribobio | 5’- GCTAGCTTGGACATCCTTA -3’ |
| Human lncRNA29Rik | Ribobio | 5’- CCTGCTTCTCCATAAGTTT -3’ |
| Other |  |  |
| Ampicillin | Sigma | Cat: BP021 |
| Vancomycine | Sigma | Cat: V2002 |
| Neomycin sulfate | Sigma | Cat: N6386 |
| Metronidazole | Sigma | Cat: M3761 |
| DMEM | Gibco | Cat:11965118 |
| FBS | Gibco | Cat:10099141 |
| HBSS | Gibco | Cat:14170161 |
| Percoll | Solarbio | Cat: P8370 |
| pcDNA™3.1/V5-His TOPO® TA  Expression Kit | Invitrogen | Cat: K4800-40 |
| FAM FLICA™ Caspase-1 Kit | Bio-rad | Cat: ICT097 |
| Pierce™ Magnetic RNA-Protein  Pull-Down Kit | Thermo Fisher Scientific | Cat:20164 |
| DIG Northern Starter Kit | Roche | Cat:12039672910 |
| Pierce™ Protein G Agarose | Thermo Fisher Scientific | Cat: 20397 |
| LDH Assay Kit | Abcam | Cat: ab102526 |
